# Virtual screening of phytochemical compounds as potential inhibitors against SARS-CoV-2 infection

**DOI:** 10.1186/s43088-021-00095-x

**Published:** 2021-01-28

**Authors:** Ram Kothandan, Cashlin Anna Suveetha Gnana Rajan, Janamitra Arjun, Rejoe Raymond Michael Raj, Sowfia Syed

**Affiliations:** Bioinformatics Laboratory, Department of Biotechnology, Kumaraguru College of Technology, Coimbatore, India

**Keywords:** In silico drug design, Virtual screening, Retroviral infection

## Abstract

**Background:**

The present pandemic situation due to coronavirus has led to the search for newer prevention, diagnostic, and treatment methods. The onset of the corona infection in a human results in acute respiratory illness followed by death if not diagnosed and treated with suitable antiretroviral drugs. With the unavailability of the targeted drug treatment, several repurposed drugs are being used for treatment. However, the side-effects of the drugs urges us to move to a search for newer synthetic- or phytochemical-based drugs. The present study investigates the use of various phytochemicals virtually screened from various plant sources in Western Ghats, India, and subsequently molecular docking studies were performed to identify the efficacy of the drug in retroviral infection particularly coronavirus infection.

**Results:**

Out of 57 phytochemicals screened initially based on the structural and physicochemical properties, 39 were effectively used for the docking analysis. Finally, 5 lead compounds with highest hydrophobic interaction and number of H-bonds were screened. Results from the interaction analysis suggest Piperolactam A to be pocketed well with good hydrophobic interaction with the residues in the binding region R1. ADME and toxicity profiling also reveals Piperolactam A with higher LogS values indicating higher permeation and hydrophilicity. Toxicity profiling suggests that the 5 screened compounds to be relatively safe.

**Conclusion:**

The in silico methods used in this study suggests that the compound Piperolactam A to be the most effective inhibitor of S-protein from binding to the GRP78 receptor. By blocking the binding of the S-protein to the CS-GRP78 cell surface receptor, they can inhibit the binding of the virus to the host.

**Supplementary Information:**

The online version contains supplementary material available at 10.1186/s43088-021-00095-x.

## Background

COVID-19, an infectious disease caused by Severe Acute Respiratory Syndrome Corona Virus-2 (SARS-CoV-2) has become an unexpected threat to the human population. With the exponential raising of the infection by followed increasing mortality rate day by day, the World Health Organization ((WHO)) has reported active cases 14,348,858 and death of 603,691 humans on 20th July 2020 [1]. The threat has forced a major responsibility to scientific society in the search for new diagnostic methods, treatment, and preventive solutions. Existing repurposed drugs have been neglected by the medical community due to associated side effects [2].

**Fig. 1:**
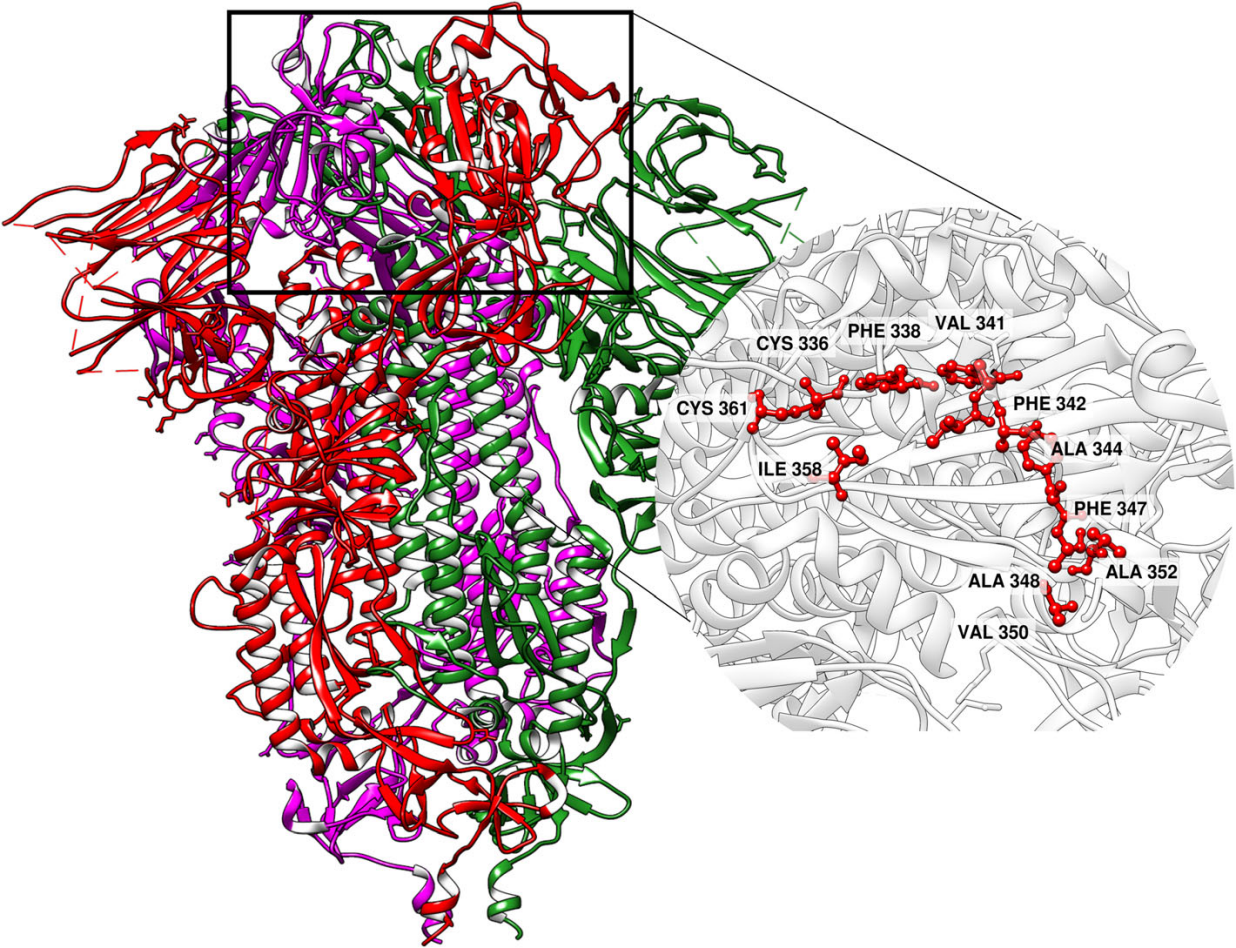
3D structure of 6X6P (Spike Protein, Trimeric structure) with chain A (Red), chain B (green), and chain C (magenta). Binding region 1 with its amino acids is highlighted

CoV belongs to the family *Coronaviridae* with a large RNA genome of 30 kb, positive-sensed and non-segmented [3]. The virion structure contains four parts of protein which are Spike proteins (S-proteins), membrane proteins (M-proteins), envelope proteins (E-proteins), and nucleocapsid proteins (N-proteins). Of these S-proteins play a major role in transfer of virion particles from the virus to the host cells using ACE2 receptor [4]. Although various literature has highlighted the importance of other proteins; the S-protein is currently well studied and has been as a potent drug target [5]. The S-protein exhibit in a homo-trimeric state with N-terminal glycosylation in which the S-polypeptide chain cleaved into two subunits S1 and S2 (Fig. 1) [3]. The S1 subunit with the larger receptor binding domain and the S2 subunit which induces the membrane fusion polypeptides forming the six helix bundle which results in complete fusion and insertion of viral genome to the host cell cytosol [6]. Hence, structural analysis on the S-protein could reveal possible mechanisms by which the viral replication could be terminated/controlled [7].

Till date, many studies are involved in targeting ACE2 receptor (angiotensin converting enzyme-2), an amino peptidase which found to act as a cellular gateway for the entry of viral RNA into the cells using the S-proteins present in the surface of the virus [5, 8]. But ACE2 receptor is not only the gateway present on cell membrane to carry this virion into the cells, there are other cellular surface proteins which could also take this role. One such protein is glucose regulated protein 78 (GRP78) residing in lumen of endoplasmic reticulum (ER), a heat shock protein with a molecular weight of 78 kDa and a molecular chaperone present in all eukaryotic cells. The major role of GRP78 is protein folding, unfolding, and resisting aggregation of proteins in cytosol [9]. The ER-protein when overexpressed escape the KDEL motif receptors which responsible for retention in ER due to its saturation or down regulation and get translocated in the cell membrane and act as cell surface GRP78 receptor (CS-GRP78 receptor) which pose the potential threat to our system for viral entry. Hypoxia, glucose starvation, and tumor causes ER stress which thus upregulate the GRP78 genes and result in over production of GRP78 [10]. Misfolded protein accumulation also results in overexpression of this protein mostly reported in neurodegenerative disorders and conditions like Alzheimer, Parkinson’s disease, and prion protein disease [11].

CS-GRP78 acts as a multifunctional receptor and binds plenty of proteins and other compounds and activates several pathways which have negative impact on the cells like apoptosis [12]. Several studies reveal that this protein has been responsible for many types of viral entry. In entry of CoxsackieVirus A9 CS-GRP78 involved as the co-receptor along with MHC Class 1 molecule on cell surface [13]. GRP78 studies reported an important host cell factor in the entry of Japanese Encephalitis Virus into the host cells [14]. Studies report that along with Dipeptidyl-peptidase 4 (DPP4), CS-GRP78 involved in the entry of MERS-CoV and reported that introduction of MERS-CoV into cells upregulate the GRP78 genes. Upregulation of GRP78 genes results in overproduction of GRP78 which help in both viral entry and development [15]. Conditions in which the primary receptor expression in host is low CS-GRP78 act as the main alternate door for the viral entry. Thus, inhibiting or reducing CS-GRP78 mediated entry of SARS-CoV-2 is essentially equivalent to inhibiting the ACE2 mediated receptor entry [16]. Protein-protein docking studies established the possible binding site where these two proteins could form complex with good HADDOCK Score, PRODIGY Binding affinity, H-bonds, and hydrophobic interaction. It would be a good approach, if we could block the region of S-protein which is responsible for binding to the CS-GRP78 receptor.

Considering the pandemic situation, several health organization, and nations in the aim of developing antiviral agents are repurposing drugs like Lopinavir (HIV), Hydroxychloroquinone (Malaria) against COVID [17, 18]. In general, repurposed pose variable target mechanisms and lack effectiveness for coronavirus strain. On the other hand, antiviral drugs also exhibit adverse side effects, which directly and indirectly affect human health [19]. To overcome this shortfall, the development of plant-based drugs and treatment strategies with minimal side-effects are expected [20]. Potentially phytochemicals, which serves as an infinite resource for drug development and novel pharmacophore may be benefited as a therapeutic agent against corona viruses. Their therapeutic applications against diverse viruses can be explained by various antiviral mechanisms such as inhibition of replication process [21] and blocking the binding of virus to the host [22].

Ancient Indian traditional techniques used several plant parts to treat various infections and disease conditions [23]. Plant parts used in the treatment was experimentally proven to pose effective medicinal properties, further studies on their crude extract and compounds extracted from the plant proved that the extracts are contained with secondary metabolites like alkaloids, flavonoids, phenols, and chalcones and were termed as phytochemicals. A large number of phytochemicals pose antimicrobial, antiviral, and antioxidant properties [24]. These plant parts which were used to treat the disease or infection were later experimentally proven for their medicinal properties [25]. Several literature studies and promising diverse biological assays reveal that a large amount of phytochemicals can be utilized as an effective treatment for retroviral infections. Recently, phytochemicals are used as an inhibitory material for viral infection including HIV, influenza, common cold, etc. [26, 27].

In this study, an attempt has been made to virtually screen potential phytochemicals with high effectiveness particularly from plants originated from Western Ghats, India. Intensive literature survey has been made prior to the screening of phytochemicals. Effectiveness of the screened compounds were validated based on the in silico docking technique by binding to the S-protein of SARS-CoV-2 and ranked based on the interaction analysis between the protein-receptor complex.

## Methods

### Computational platform

All computational analyses were performed on Linux Mint 18 platform in a Dell T20 server on Intel Xeon Quad Core 3.2 Hz.

### Receptor preparation

The SARS-CoV-2 S-protein (PDB ID: 6X6P at 3.2 *Å*) with the spike glycoprotein was downloaded from RCSB Protein Bank (PDB). The protein is a trimeric viral fusion protein with subunits S1 and S2. S1 contains the receptor binding protein (RBD) responsible for host cell receptor binding and the S2 subunit facilitates the membrane fusion between the viral and host cell membranes. The viral protein contains 1274 amino acids and weighs 434.87 kDa. For the purpose of the study, we utilized only Chain A from the S-protein for docking analysis. Protein visualization and manipulation of the structures was done using UCSF Chimera [28].

### Binding-site preparation

The prediction of binding site was done with respect to the available literature [29] and then verified based on the presence of hydrophobic regions on the surface of the protein. For the purpose of the study, we utilized the same binding site between the S-protein and CS-GRP78, since our main aim is to block the binding of S-protein to the ER protein [29]. The hydrophobicity of the protein is calculated using GRAVY server [30]. The S-protein contains four hydrophobic regions in the receptor binding domain (RBD) ranging from 318 to 510 as of the S1 subunit. The binding energy values obtained from protein binding energy prediction (PRODIGY) [29].

### Screening for phytochemicals

Phytochemicals with antiviral, antimicrobial, and antioxidant properties were screened based on the previous literature and were sorted, filtered based on their geographical origin [31–33]. More than 100 compounds were initially screened. 3D and 2D structures were downloaded from PUBCHEM database [34] and drugbank.ca [35] in both SDF and Simplified Molecular Input Line Entry System (SMILES) format [36] for further screening procedures. In order to select the potential drug-like compounds, several filters were applied on the structures.

Initially, LIPINSKI rule of five (RO5) was applied to screen potential drug-like compounds [37]. Drug-like compounds screened from RO5 was further fed into SWISS-ADME server for screening based on the pharmacokinetics, drug-likeliness, and medicinal chemistry friendliness of small molecules [38]. In addition, the physicochemical properties and the drugability of the selected compounds were also predicted with SWISS-ADME server. Hydroxychloroquinone (HCQ) (PUBCHEM ID: 3652), a well-known retro viral drug was used as a standard throughout the studies [39].

### Multiple ligand docking

For the purpose of study, Autodock was employed for docking simulations [40]. Initially, water molecules and hetero atoms were removed from the structures [41]. Addition of gasteiger charges and H-bond was carried out prior to the docking protocol. A grid box of 25 *×* 22 *×* 19 *Å* was assigned on the surface of the receptor with respect to the identified active site. In this present study, multiple ligands have been screened against the receptor. Multiple ligand docking was carried out using PyRx in the Autodock environment [42]. The docked structures were analyzed for root mean square deviation (RMSD), lowest energy conformer, and hydrogen bond (HB) interaction. 2D protein-ligand interaction profile was obtained using Protein Plus Server [43, 44] and Protein Ligand Interaction Profiler [45].

### Physicochemical property of the bound ligand

To validate the bound ligand with the receptor, the physicochemical and pharmacokinetic properties of the drug are analyzed using SWISS-ADME server [38]. The toxicity profile is analyzed using admetSAR 2.0 [46], which provides information on the ADMET properties. Screened compounds were subjected to Ghose filter [47] and Muegge Filter [48].

## Results

In the present study, 57 phytochemicals were initially screened based on their structural and physicochemical properties, later only 39 compounds were utilized for the docking analysis based on the various filters utilized during the course of the study. Binding sites were initially chosen based on the literature survey made on the target protein 6X6P. Potential binding sites were further selected based on the hydrophobic index obtained from GRAVY server. Results from GRAVY server indicate significant values for the four-binding region R1-R4 as − 0.24, − 0.3, − 0.28, and − 0.08, respectively. Binding sites were also considered based on their predicted binding energy by PRODIGY. Results from PRODIGY for the four binding regions R1–R4 are − 12.4, − 9.8, − 10.1, and – 14 kcal/mol, respectively. Region 4 (R4) [− 9.8 kcal/mol] and Region 1 (R1) [− 12.4 kcal/mol] of the S-protein pose a higher hydrophobicity. However, R1 was considered for the potential binding site.

**Fig. 2:**
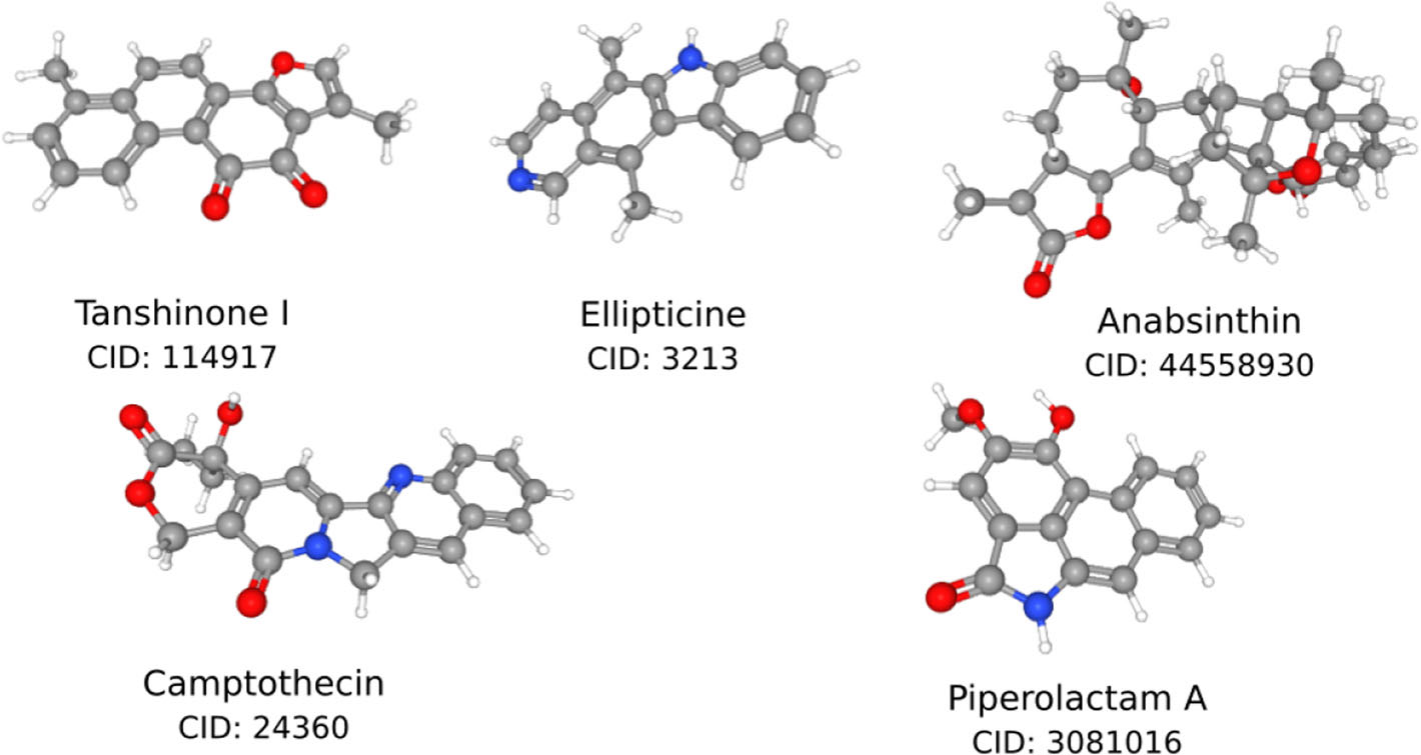
Virtually screened ligands from PubChem compounds

Prior to the actual binding, the ligands are screened based on the RO5 and SWISS-ADME filters. Among the 39 finally screened compounds, only 5 compounds exhibited docking energy values above − 8.9 to 8.3 kcal/mol for SARS-CoV-2 S-protein Region 1 (Fig. 2). Among them, Tanshinone I exhibited an average docking score of − 8.9 kcal/mol and Piperolactam A with a docking score of − 8.3 kcal/mol ranked the strongest both in terms of binding energy and HB interaction. A list of all other ranked ligands based on the binding energy is listed in the Table 1. The standard ligand, HCQ resulted in a docking score of − 4.7 kcal/mol and was used to compare other phytochemicals docked with the S-protein.

**Table 1:** Dock Scores obtained from Autodock Pyrex of Screened Ligands

Ligand	Binding energy (kcal/mol)	Molecular weight (g/mol)	Rotatable bonds	HB acceptor	HB Donar	TPSA ( Å^2^)
Tanshinone I	-8.9	276.29	0	3	0	47.28
Ellipticine	-8.4	246.31	0	1	1	28.68
Anabsinthin	-8.4	496.64	0	6	1	82.06
Camptothecin	-8.3	348.35	1	5	1	81.42
Piperolactam A	-8.3	265.26	1	3	2	62.32

**Fig. 3:**
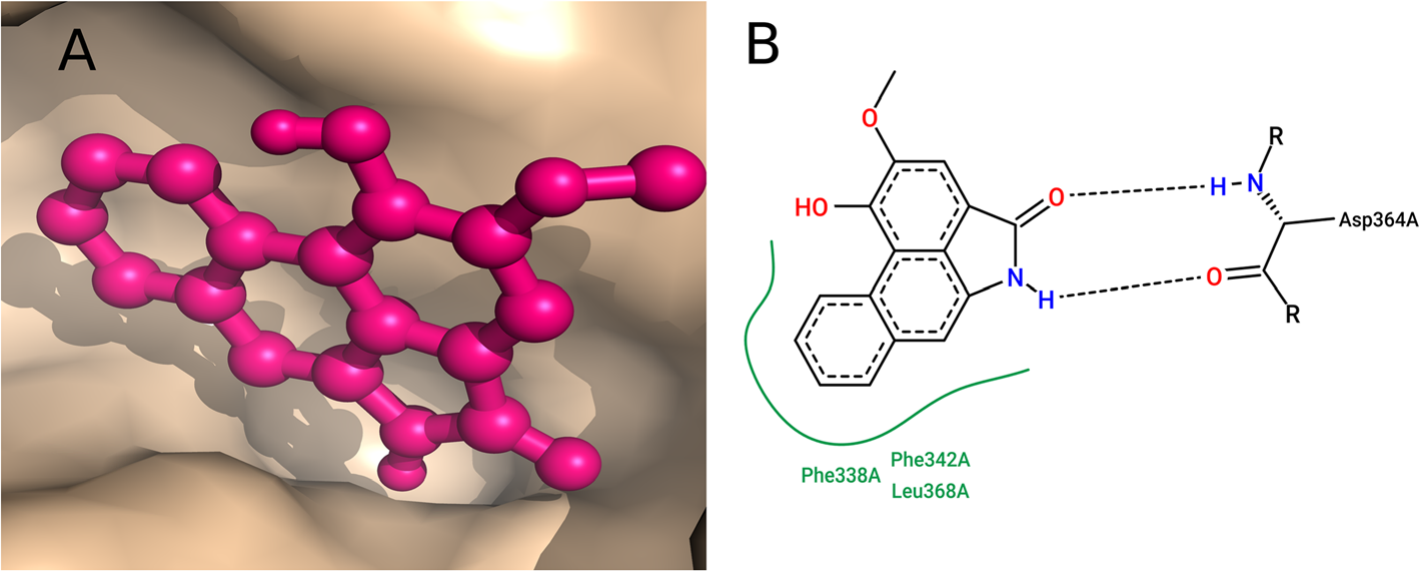
Piperolactam A docked with S-protein. **a** Surface image of Piperolactam A docked with the Region 1 of 6X6P S-protein. **b** 2D Interaction map of the Piperolactam A with the S-protein. Interaction profiles indicate two H-bond with ASP364

The HCQ-S-protein complex has two HB interactions with ARG355 and TYR396 which contribute to the hydrophobic interactions. In comparison with HCQ, Tanshinone I has the highest binding affinity of − 8.9 kcal/mol compared to all other compounds and has one HB of distance 2.38 *Å* with the acceptor atom O2 of ASN343. Tanshinone I also formed a good hydrophobic interaction with the neighboring amino acids *viz.,* PHE338, PHE342, VAL367, LEU368, and PHE374 (Table 2), whereas the second lead compound Piperolactam A with binding affinity − 8.3 kcal/mol is found to form two HB interaction with the S-protein in which both bonds are from ASP364 to the compound with a distance of 1.99 *Å* and 2.05 *Å  *(Fig. 3).

**Table 2:** HB Interaction with the Screened Ligands

Ligands	Hydrogen bonds	Hydrogen Bond Interaction	Distance H-A (A^2^)
Tanshinone I	1	ASN A: 343	2.3
Ellipticine	0	-	-
Anabsinthin	1	ASP A:364	2.68
Camptothecin	2	GLY A:339, ASP A:364	2.83,3.21
Piperolactam A	2	ASP A:364, ASP A:364	1.99, 2.05
Hydroxychloroquine	2	355 A:ARG,466 A:ARG	2.27,2.37

Compounds with good ADME parameters are suitable for the drug discovery process. Analysis of the lipophilicity of the screened compounds showed positive values which indicates enhanced rate of adsorption [49] as described in supplementary material.

## Discussion

In this present study, although during the selection and validation of the predicted binding sites, both Region R1 and R4 resulted as potential binding sites among the four predicted sites. Based on the hydrophobicity index, R4 is marked with higher index values; however, the site fails to show minimum interaction with the known receptor CS-GRP78 [10]. The binding affinity of R4 is – 14 kcal/mol which is much lower than the R1 with − 12.4 kcal/mol. Based on the binding affinity values, R1 is considered to be a potential binding site for the screened ligands. The binding site involves the residues of ASN343, ASP364, GLY339, ARG355, and ARG466. Additional amino acids at the active site are PHE338, VAL367, LEU368, PHE342, PHE337, and TYR396 (Fig. 1).

With respect to screened ligands, the hydrophilicity factor (LogS) of Camptothecin (− 3.49) and Piperolactam A (− 3.87) showed higher LogS values (high permeation and hydrophilicity) than the other screened compounds [50]. Among the screened compounds, Ellipticine (− 5.05) showed a less LogS value indicating a poor permeation. Similarly, anabsinthin acts a good substrate for P-glycoprotein, which is highly susceptible to changes in pharmacokinetics by acting as either P-glycoprotein inducer or inhibitors [51]. Thus, anabsinthin could cross the blood-brain barrier (BBB) and it may render the compound functionally inactive [52]. Further, anabsinthin could not satisfy Ghose rule [47] of drug-likeness with three violations and Muegge rule [48] of drug-likeness with one violation. Piperolactam A showed a good pharmacokinetics property with good GI absorption, cross BBB, affects only two cytochrome P450 enzymes compared to others and is also not a substrate for P-glycoprotein. The phytochemical compound Piperolactam A has synthetic accessibility of 2.02 which makes it at ease of synthesis of the compound in spite of an alert in the Brenk filter. Whereas Camptothecin was found with no violations but its synthetic accessibility is of higher value than that of Piperolactam A. Toxicity analysis is essential for a drug to know the potential hazard to be produced by it. The predicted toxicity profile from our study reveals that all the compounds are relatively safe.

Results from the docking analysis suggest that the Piperolactam A pocketed well with good hydrophobic interaction with residues PHE338, PHE342, and VAL367as well  (Fig. 3). Based on the results obtained, the study suggests use of Piperolactam A as a potential antiretroviral drug against SARS-CoV-2. Piperolactam, an aristolactam isolated from Piper betle Linn and its abundant availability in Western Ghats [53].

## Conclusion

In this study, five phytochemical compounds with antiretroviral properties were screened against the receptor 6X6P. These compounds displayed appreciable pharmacokinetic and physicochemical properties. ADMET analyses of these compounds reveal that the compound Piperolactam A from *Piperaceae* family to be most effective in the inhibition of S-protein from binding to the CS-GRP78 receptor.

## Supplementary Information

**Additional file 1.**

## Data Availability

All data used in the study is given as supplementary data.

## Abbreviations

ACE2: Angiotensin converting enzyme-2
ER: Endoplasmic reticulum
DPP4: Dipeptidyl-peptidase 4
CS-GRP78: Cell surface glucose receptor protein 78
HIV: Human immuno-deficiency virus
PRODIGY: Protein energy binding prediction
SMILES: Simplified Molecular Input Line Entry System
BBB: Blood-brain barrier
