# Supplementary material for: Virtual screening of phytochemical compounds as potential inhibitors against SARS-CoV-2 infection
Source: Beni Suef Univ J Basic Appl Sci. 2021 Jan 28;10(1):9. doi: 10.1186/s43088-021-00095-x (PMC7841026; doi:10.1186/s43088-021-00095-x)
Supplement: Supplementary file 1 — Additional file 1. [file 43088_2021_95_MOESM1_ESM.pdf]

Supplementary Data

Additional file 1 — Binding Affinity values for PROGIDY and Hydrophobicity Values from GRAVY

Regions	Hydrophobicity index (GRAVY)	Hydrophobic interactions (in numbers)	Binding affinity(kcal/mol) PRODIGY
Region 4	-0.08	10	-14
Region 1	-0.24	7	-12.4
Region 3	-0.28	4	-10.1
Region 2	-0.3	3	-9.8

Additional file 2 — Filters Applied to Screen Lead Compounds and their respective output

Ligand	Lipinski	Ghose	Veber	Egan	Muegge	Bioavailability score
Tanshinone I	Yes	Yes	Yes	Yes	Yes	0.55
Ellipticine	Yes	Yes	Yes	Yes	Yes	0.55
Anabsinthin	Yes(0)	No(3)	Yes	Yes	No(1)	0.55
Camptothecin	Yes(0)	Yes	Yes	Yes	Yes	0.55
Piperolactam A	Yes(0)	Yes	Yes	Yes	Yes	0.55

Additional file 3 — Scores from SWISS ADME Server

Compounds	Swiss ADME cLogP	Mean Calculated cLogP	Lipophilicity (Consensus Log Po/w)	Log S (ESOL)
Tanshinone I	3.4	3.615	3.4	-4.41
Ellipticine	3.92	4.1	3.92	-5.05
Anabsinthin	3.64	3.455	3.58	-4.59
Camptothecin	2.2	2.115	2.2	-3.49
Piperolactam A	2.85	3.125	2.85	-3.87

Additional file 4 — Lead Likeliness Values for the Screened Compounds

Ligand	PAINS	Brenk	Leadlikeness	Synthetic Accessibility
Tanshinone I	2	1	No(1)	3.28
Ellipticine	0	0	No(2)	1.6
Anabsinthin	0	2	No(1)	7.08
Camptothecin	0	0	Yes	3.84
Piperolactam A	0	1	Yes	2.02

Additional file 5 — Toxicity Analysis of the Screened Compounds

Ligand	hERG inhibition	AMES mutagenicity	Carcinogenicity	Acute Oral Toxicity
Tanshinone I	-	+	-	III
Ellipticine	-	+	-	III
Anabsinthin	-	-	-	I
Camptothecin	-	-	-	II
Piperolactam A	-	+	-	III
